# Stimulated Raman Histology for Intraoperative Guidance in the Resection of a Recurrent Atypical Spheno-orbital Meningioma: A Case Report and Review of Literature

**DOI:** 10.7759/cureus.5905

**Published:** 2019-10-14

**Authors:** Evan Luther, Alejandro Matus, Daniel G Eichberg, Ashish H Shah, Michael Ivan

**Affiliations:** 1 Neurological Surgery, University of Miami Miller School of Medicine, Miami, USA; 2 Neurological Surgery, Florida International University, Herbert Wertheim College of Medicine, Miami, USA

**Keywords:** stimulated raman histology, brain tumor, atypical meningioma, tumor margins, oncology, neuro-oncology, skull base, pathology, craniotomy, neurosurgery

## Abstract

Meningiomas are the most common intracranial, extra-axial neoplasms and account for a significant proportion of all central nervous system (CNS) tumors. Regardless of the grade, treatment typically involves upfront surgical resection. However, in many instances, especially in meningiomas arising from the skull base, complete removal is often difficult given the close proximity to important anatomic structures. In this report, we discuss the use of stimulated Raman histology as a means to identify tissue boundaries during the resection of an extensive, recurrent, atypical spheno-orbital meningioma.

We report a 75-year-old male with a history of a prior left frontotemporal craniotomy for a grade II meningioma three years prior, who presented with worsening left-sided visual loss and pronounced temporal bossing. Repeat magnetic resonance imaging (MRI) revealed a recurrent left spheno-orbital tumor suggestive of a meningioma extending into the middle cranial fossa, the lateral orbit, and the temporalis muscle. He underwent an extended orbito-pterional craniotomy, and intraoperative stimulated Raman histology aided in the identification of tumor margins within the orbit and the temporalis muscle in order to better preserve the normal orbital contents and muscle bulk of the infratemporal fossa.

This case demonstrates the utility of stimulated Raman histology during the resection of invasive skull base tumors. The immediate intraoperative Raman histologic sections can clearly identify tissue boundaries and thus help preserve important anatomic structures. Continued development of this method can potentially improve the accuracy of intraoperative diagnoses and guide surgeons during tumor resections near eloquent anatomical regions or important normal structures.

## Introduction

Meningiomas are the most common adult brain tumor accounting for 36% of all central nervous system (CNS) neoplasms [1]. World Health Organization (WHO) grade II/III meningiomas are not benign and most clinicians agree that gross total resection should be the goal of surgery to prevent tumor recurrence [2-6]. However, the margins of these neoplasms can be difficult to identify intraoperatively and there still remains no standardized diagnostic technique to locate tumor margins during excision [7].

Stimulated Raman histology allows for the fast, high-resolution acquisition of structural information through spectral image generation [8-9]. This occurs because of a phenomenon known as the Raman effect in which a scanning laser emits photons onto a sample thus generating a scattering pattern detected via a two-dimensional microarray. When applied to biological tissue, this can create a real-time histologic image without the use of flash freezing necessary in conventional frozen sections thus significantly reducing the time required to prepare pathology results [7,10-11].

To that end, several preclinical studies have been performed using stimulated Raman histology on ex vivo meningiomas and have shown exceedingly high sensitivity and specificity when attempting to discriminate tumors from normal tissue [12-14]. Although in vitro studies have also demonstrated that stimulated Raman histology is capable of detecting brain tumor margins, there have been no reports using this technique intraoperatively [15]. Therefore, we present the first case of a patient with a recurrent WHO grade II spheno-orbital meningioma in which stimulated Raman histology was used to assist with identifying tumor margins during surgical excision.

## Case presentation

After institutional review board approval, this patient was prospectively consented for participation in this study. Patient clinical information, including demographic data, presenting symptoms, intraoperative findings, and post-surgical clinical status, were obtained from the electronic medical record.

**Figure 1:**
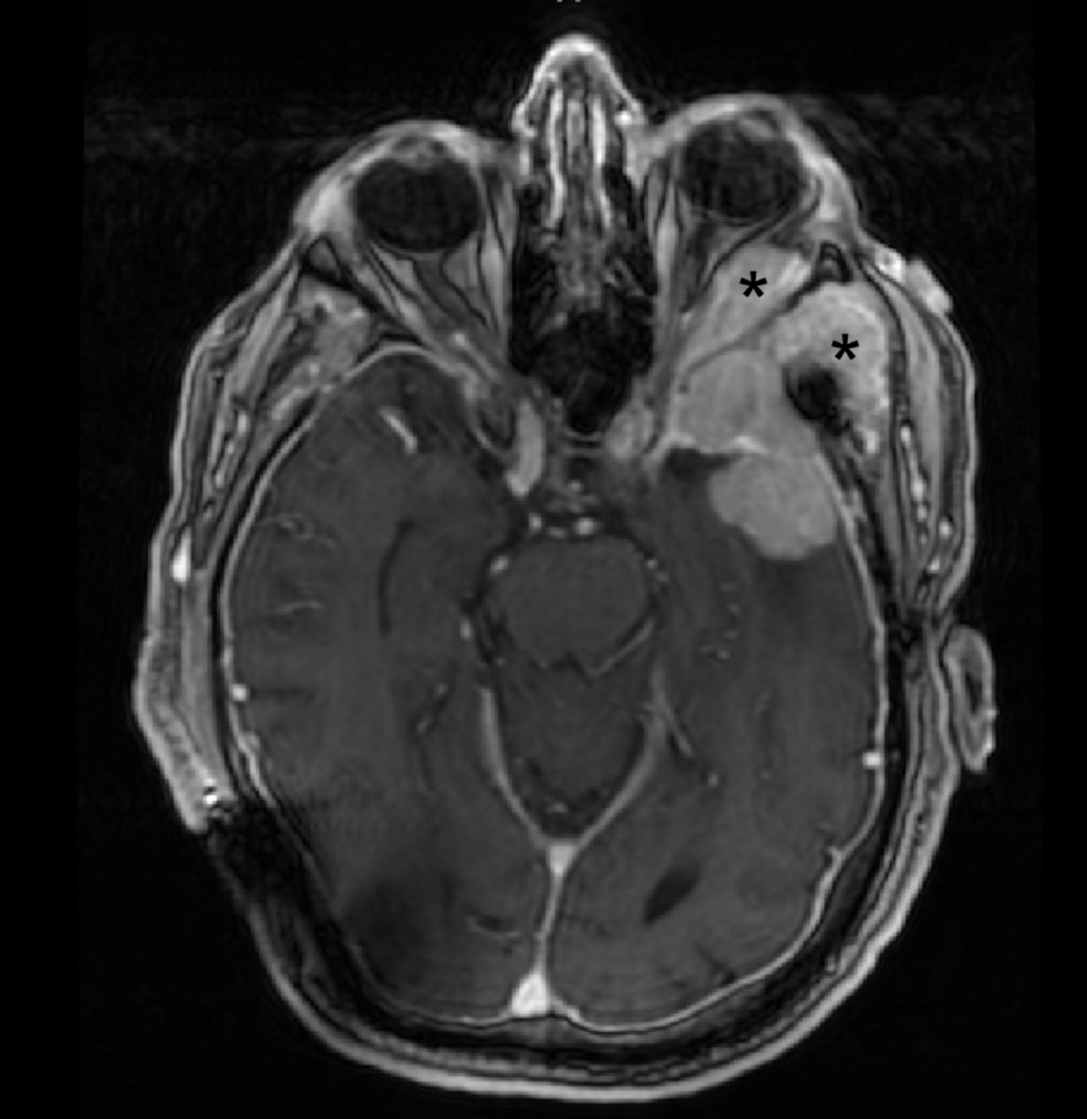
Preoperative axial MRI demonstrating tumor involvement of the lateral orbit and temporalis muscle * = Tumor invasion of the lateral orbit and temporalis muscle MRI: magnetic resonance imaging

**Figure 2:**
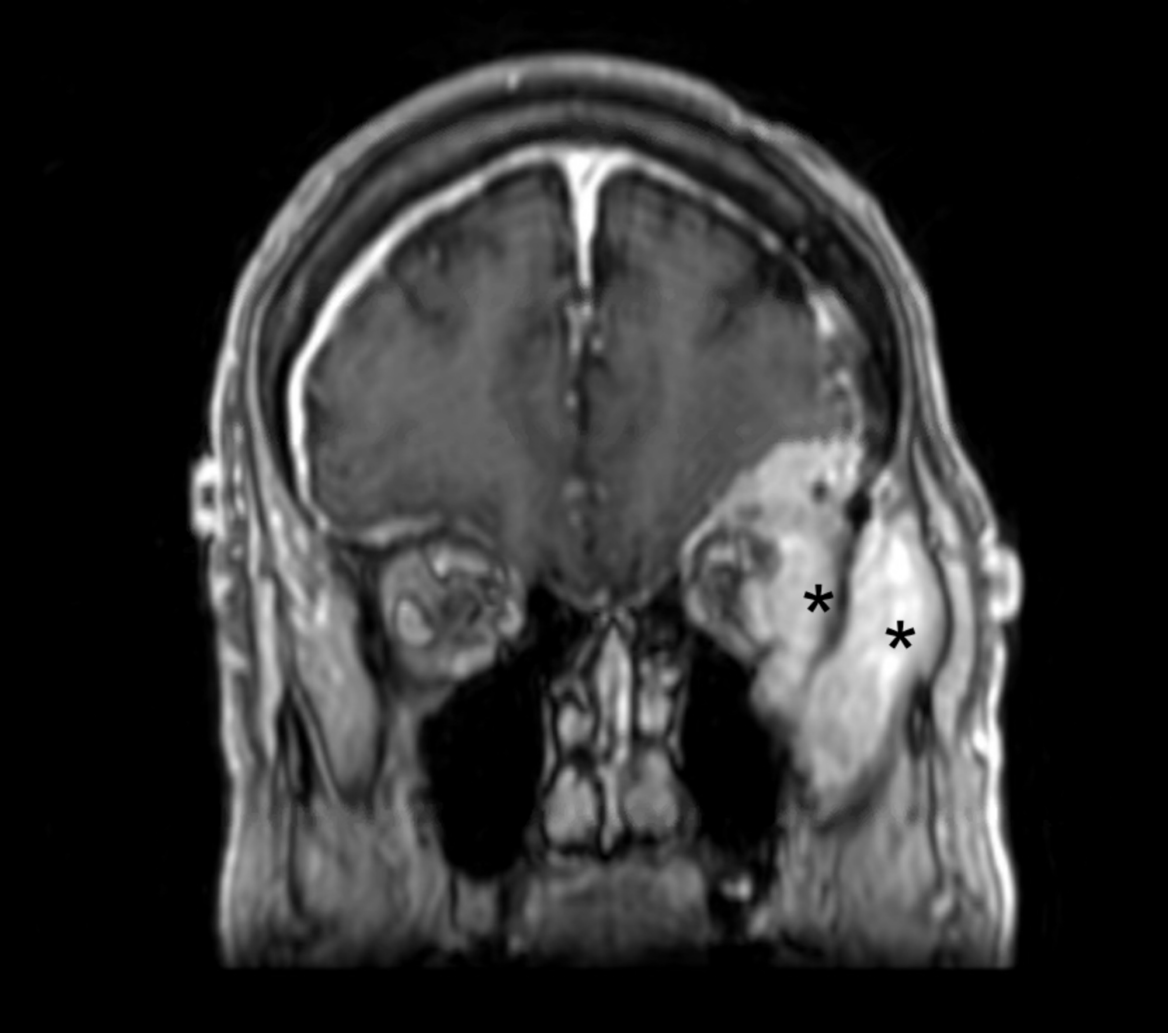
Preoperative coronal MRI demonstrating tumor involvement of the lateral orbit and temporalis muscle * = Tumor invasion of the lateral orbit and temporalis muscle MRI: magnetic resonance imaging

**Figure 3:**
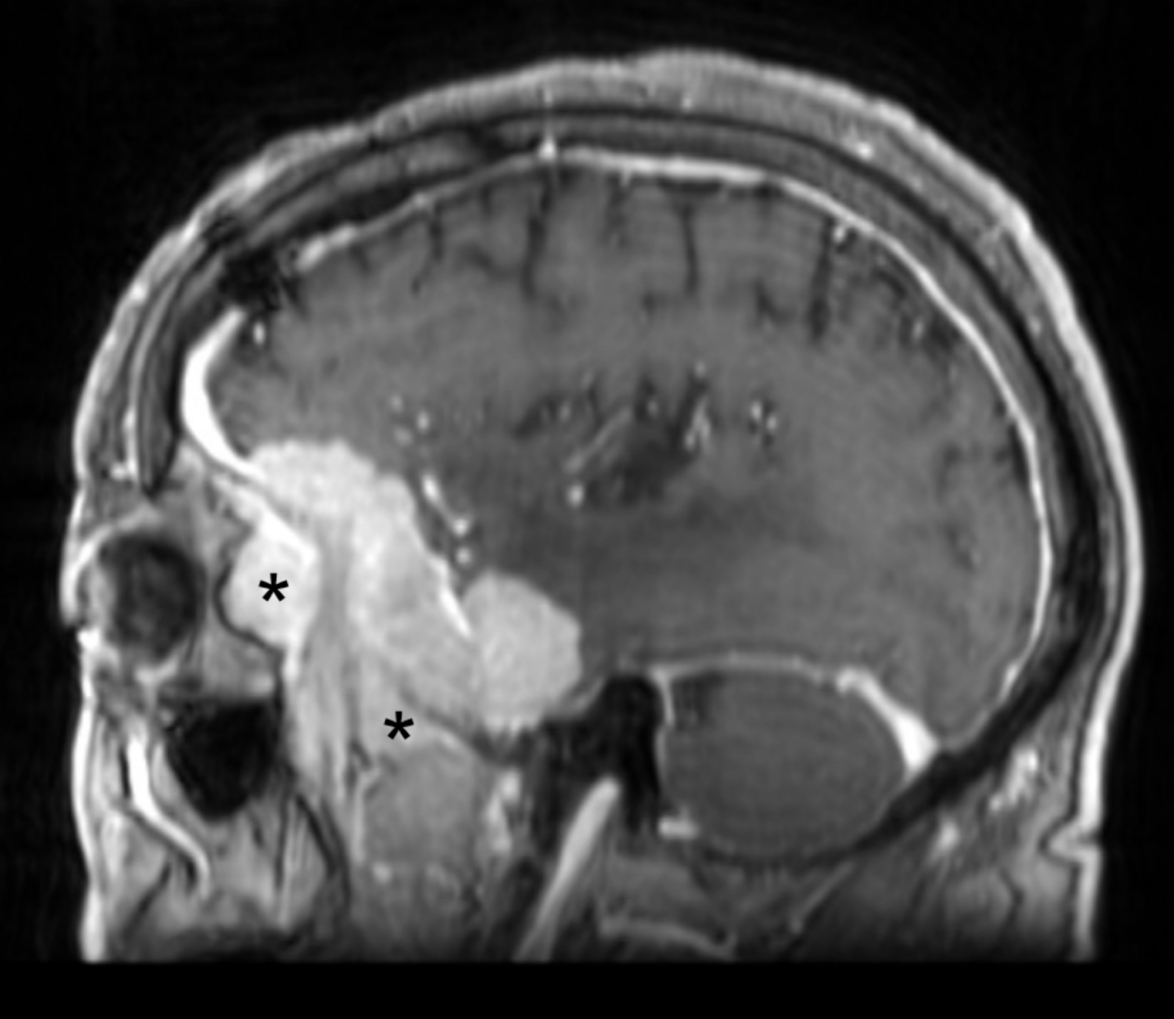
Preoperative sagittal MRI demonstrating tumor involvement of the lateral orbit and temporalis muscle * = Tumor invasion of the lateral orbit and temporalis muscle MRI: magnetic resonance imaging

A 75-year-old male underwent a left frontotemporal craniotomy in 2014 for the resection of a WHO grade II meningioma. He presented four years later with new left-sided vision loss, proptosis, diplopia, and temporal bossing. MRI demonstrated a recurrence of the lesion with invasion into the temporalis muscle and the superolateral orbital wall (see Figures 1-3). 

**Figure 4:**
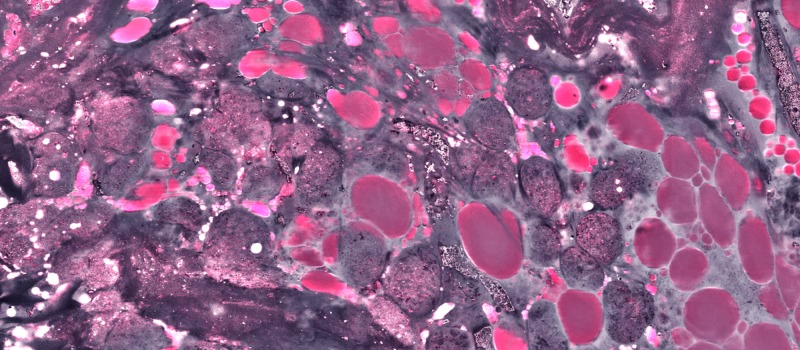
Lacrimal gland showing organized glandular tissue and lipid deposits; no tumor seen

**Figure 5:**
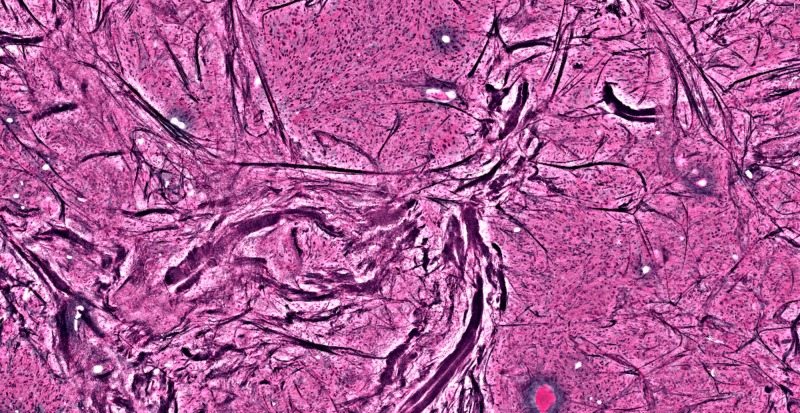
Dark streaks showing large acellular collagen fibers, representing bone fragments, with active tumor cells around it

**Figure 6:**
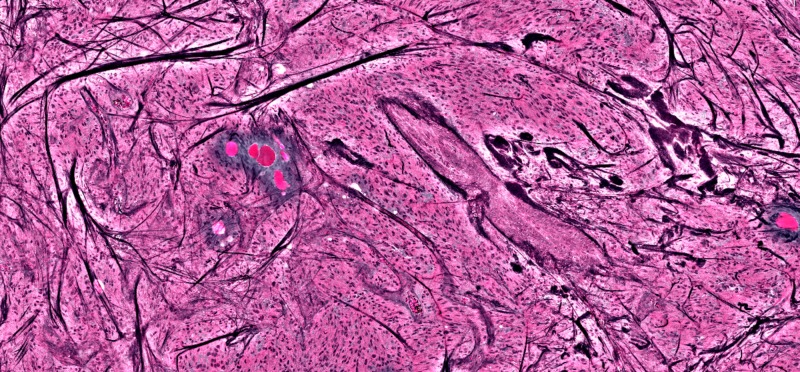
Large acellular pink areas are lipid deposits within the temporalis muscle, again with active tumor surrounding them

After induction of general anesthesia and registration of neuronavigation, a large pterional incision was made and after the dissection of the superficial temporalis fascia, the tumor was immediately identified within the temporalis muscle. The tumor was followed anteriorly until it entered the orbit. Then, after performing a superolateral orbitotomy, the tumor was identified intraorbitally back to the orbital apex and superior orbital fissure. The tumor consistency was soft, making it challenging to distinguish from the orbital fat and lacrimal gland. Furthermore, it was adherent to the temporalis muscle, making dissection difficult without removing a significant portion of the muscle. As a result, multiple samples were taken from the margin of the tumor within the temporalis/temporal bone and the orbital apex to be analyzed using stimulated Raman histology (SRH) in an effort to prevent disruption of the lacrimal gland, the superior and lateral rectus muscles, and the temporalis muscle. These specimens immediately underwent a one-step squash prep in which the fresh tissue was placed on a standard uncoated glass slide and flattened using normal saline and a coverslip. This slide was then placed on a motorized stage and focused using standard transmission light microscopy. Software was then utilized to provide high-resolution microscopic images requiring no special staining or frozen preparation. This resulted in readily available images that were subsequently used to distinguish tumor boundaries from normal lacrimal gland and muscle fibers/bone (Figures 4-6).

**Figure 7:**
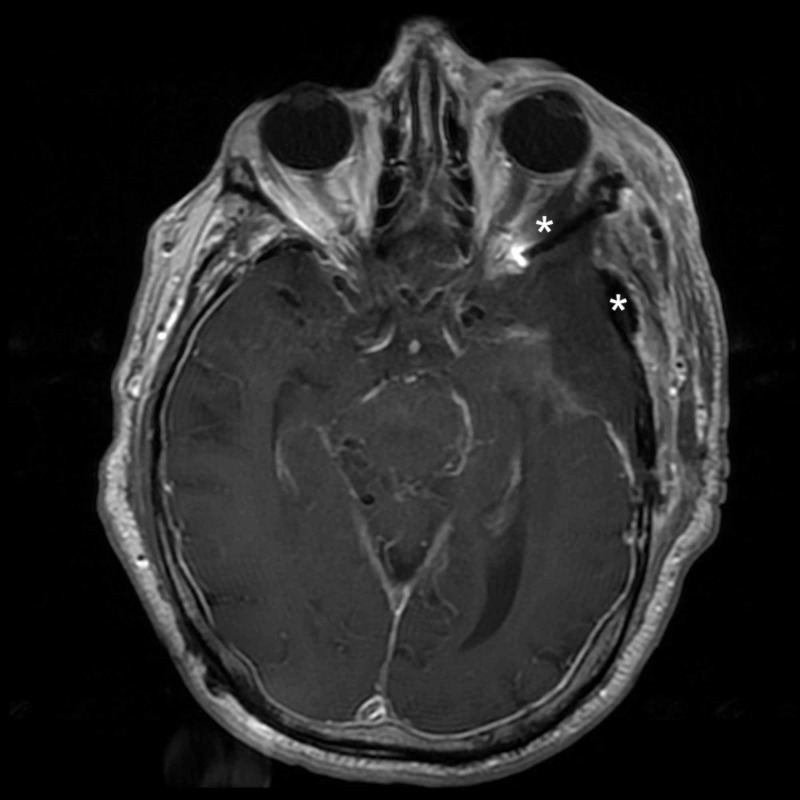
Postoperative axial MRI demonstrating resection of the intraorbital and intramuscular tumor component * = Areas of prior tumor invasion of the lateral orbit and temporalis muscle MRI: magnetic resonance imaging

**Figure 8:**
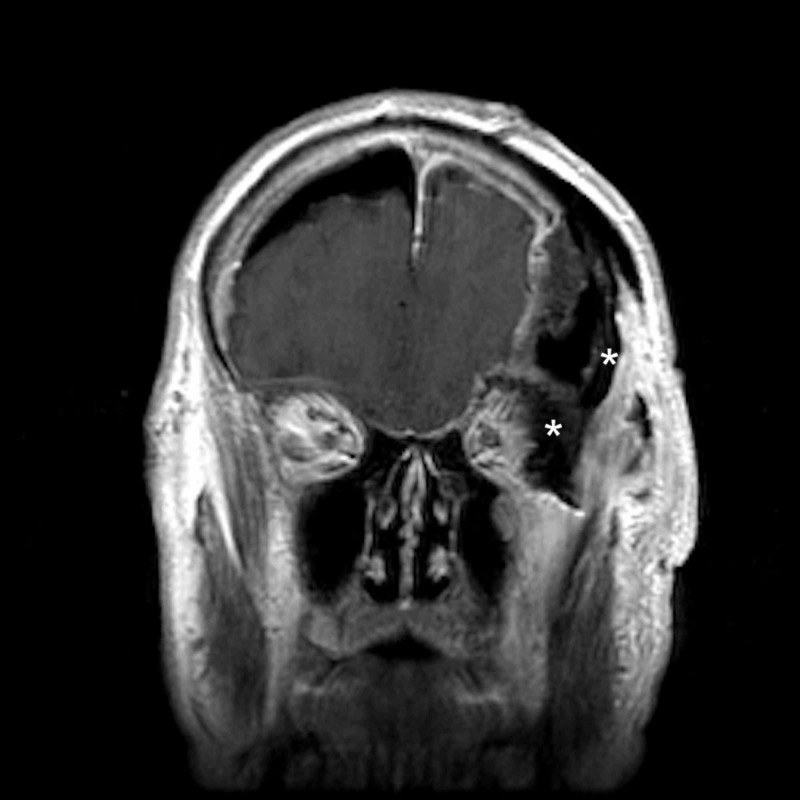
Postoperative coronal MRI demonstrating resection of the intraorbital and intramuscular tumor component * = Areas of prior tumor invasion of the lateral orbit and temporalis muscle MRI: magnetic resonance imaging

**Figure 9:**
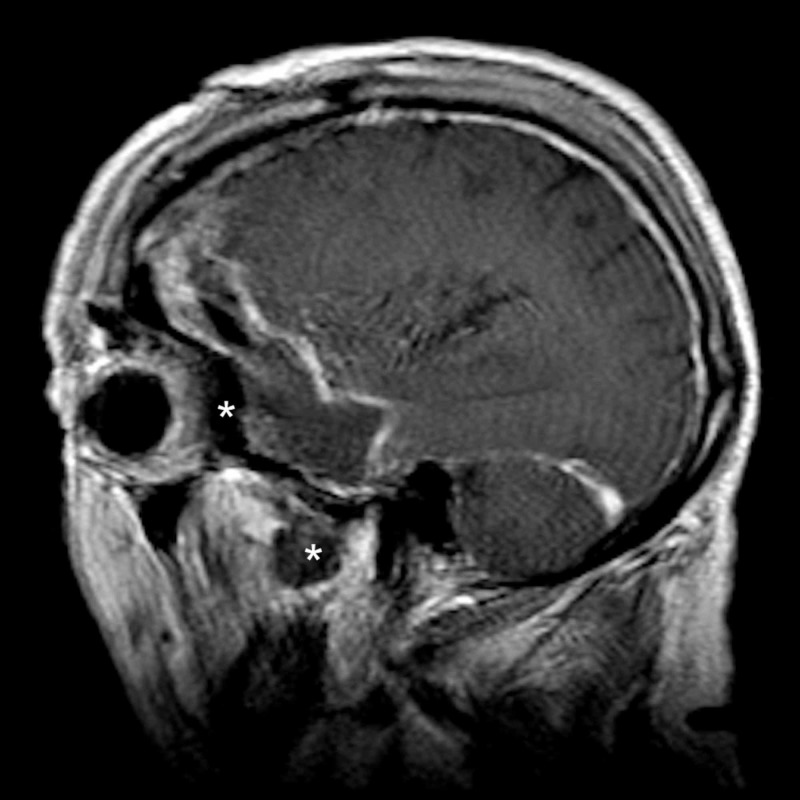
Postoperative sagittal MRI demonstrating resection of the intraorbital and intramuscular tumor component * = Areas of prior tumor invasion of the lateral orbit and temporalis muscle MRI: magnetic resonance imaging

The resection was then continued extradurally via a Dolenc-Hakuba approach to expose the cavernous sinus and middle fossa floor. With this approach, the remaining tumor was removed from the middle fossa skull base. V2 was exposed back to the trigeminal ganglion as well as the infratemporal fossa and pterygopalatine fossa without entering the maxillary or sphenoid sinuses. The intradural tumor was then resected and all the involved dura accessible with the microsurgical technique. Afterwards, SRH and the surgical microscope identified no active tumor in the orbit or temporalis muscle. A small amount of residual tumor was intentionally left within the cavernous sinus in an attempt to prevent injury to any of its contents. The surgery, including the intradural component, was uneventful and the patient had no complications with notably improved vision and diplopia by three days postoperatively. The orbit was reconstructed with mesh and bone and the dural opening was closed with a dural substitute in a watertight fashion. Postoperative MRI demonstrated good decompression of the orbit and removal of the tumor invading the temporalis muscle (Figures 7-9).

**Figure 10:**
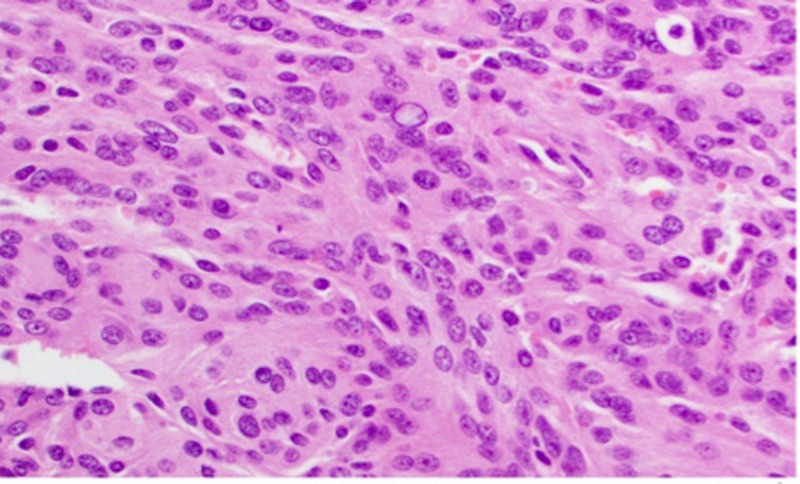
Haemotoxylin and Eosin (H&E) staining of a WHO grade II meningioma at high-power magnification WHO: World Health Organization

**Figure 11:**
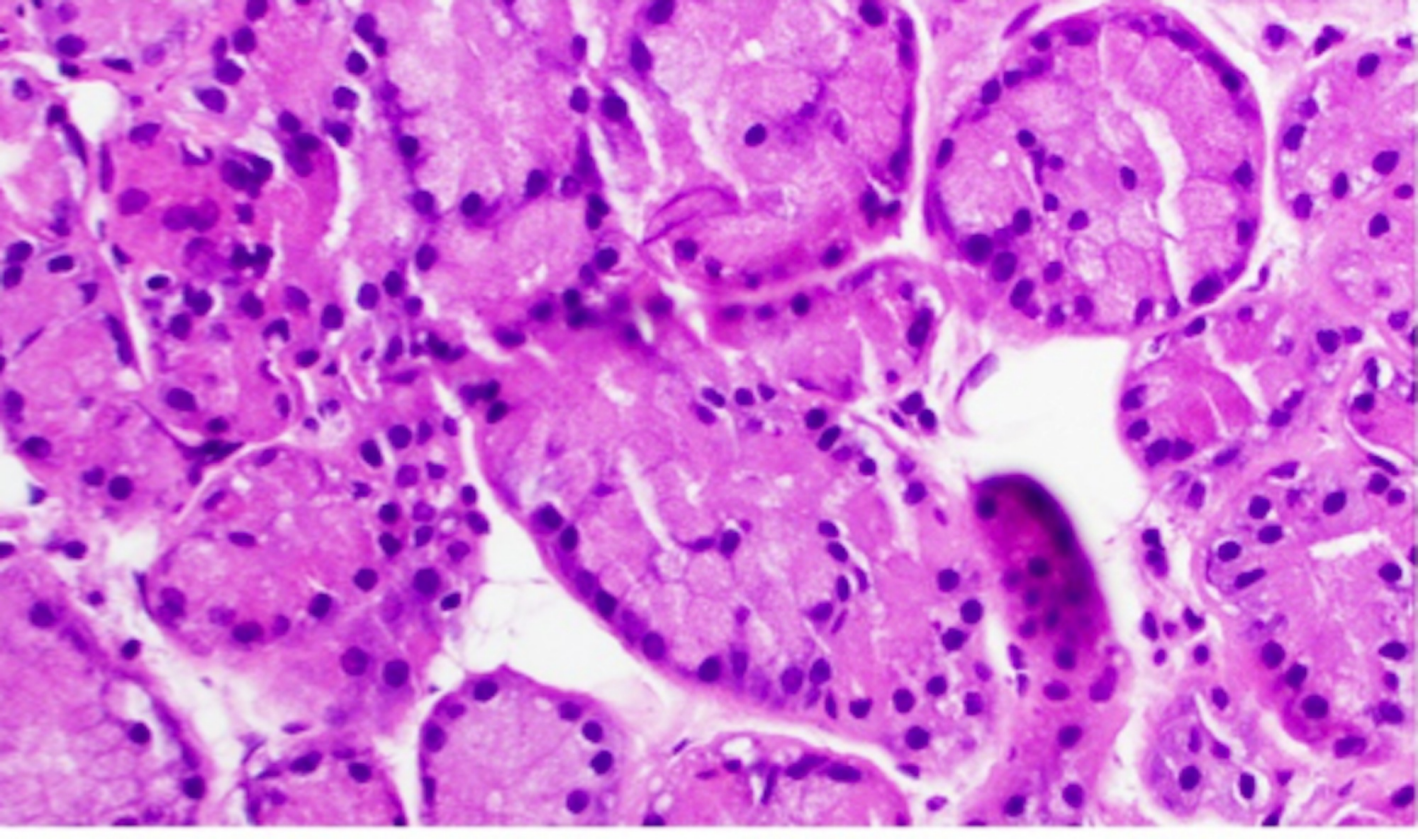
H&E staining of lacrimal gland tissue at high-power magnification H&E: Haemotoxylin and Eosin

**Figure 12:**
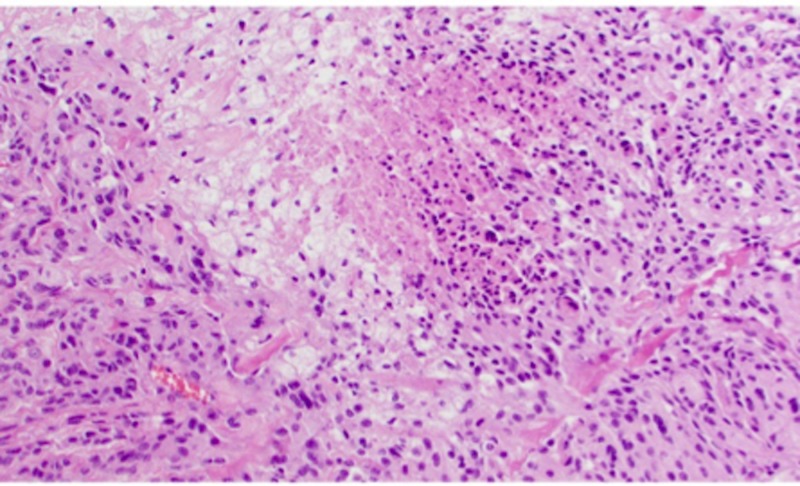
H&E staining of WHO grade II meningioma at high-power magnification; necrosis is seen in the center of the image H&E: Haemotoxylin and Eosin WHO: World Health Organization

**Figure 13:**
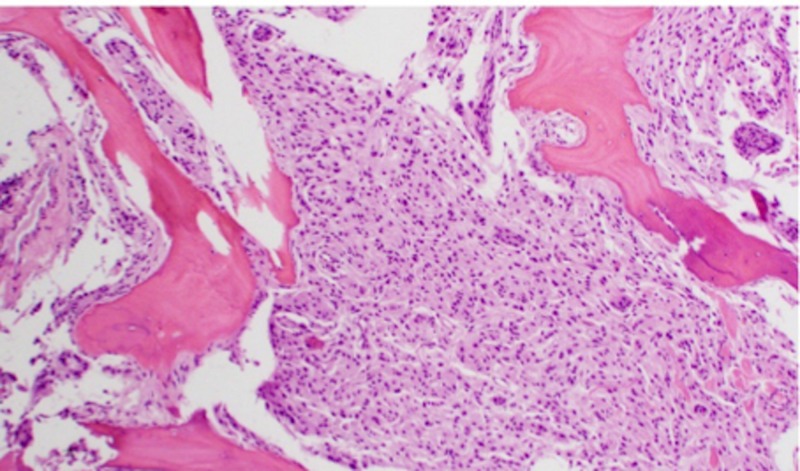
H&E staining of WHO Grade II meningioma at high-power magnification demonstrating bone invasion H&E: Haemotoxylin and Eosin WHO: World Health Organization

**Figure 14:**
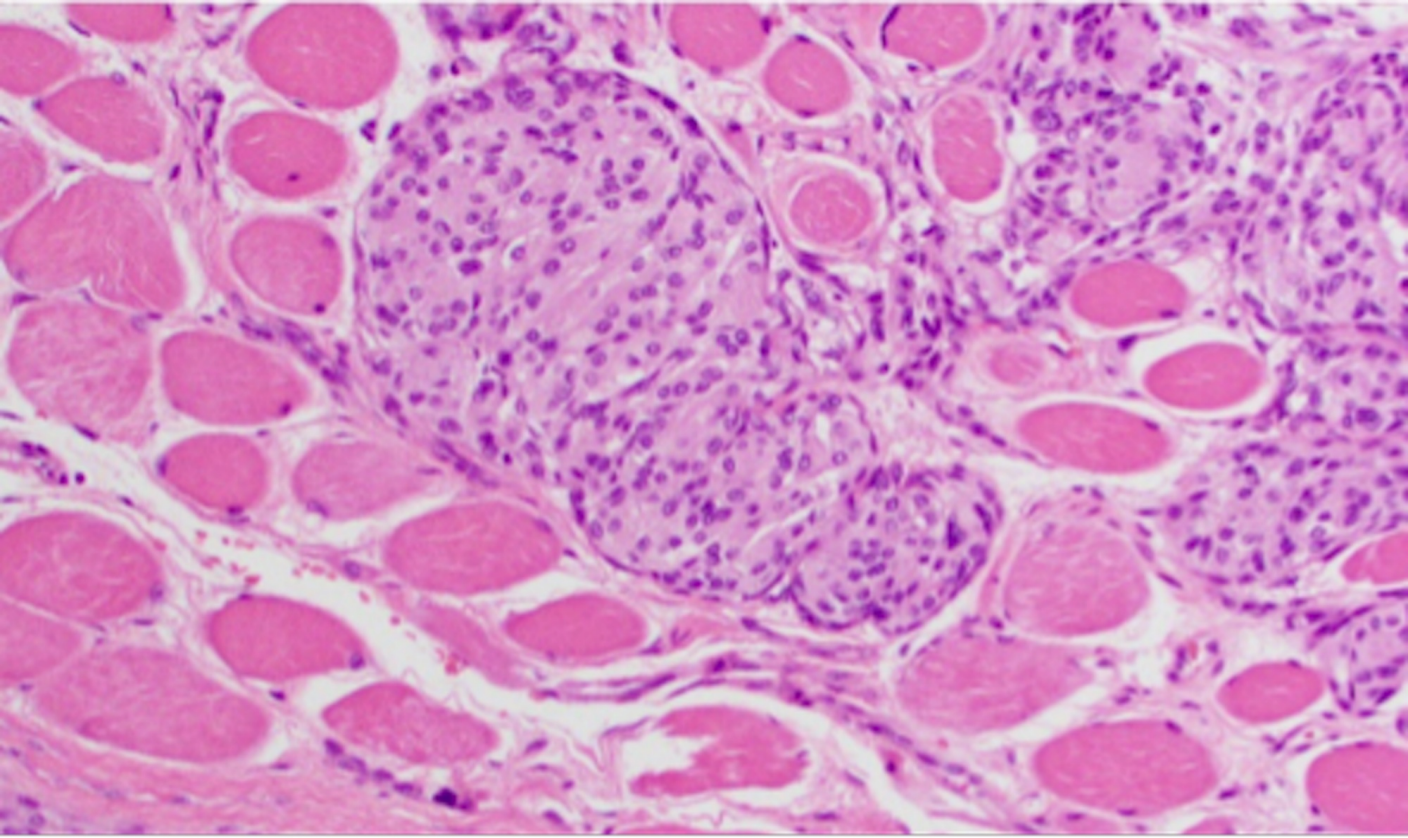
H&E staining of WHO grade II meningioma at high-power magnification demonstrating skeletal muscle invasion H&E: Haemotoxylin and Eosin WHO: World Health Organization

Histopathologic examination, finalized at two weeks postoperatively, is shown in Figures 10-14 and confirms the diagnosis of atypical (WHO grade II) meningioma. At discharge, the patient was at his neurologic baseline and at the two-week follow-up, his diplopia had resolved.

## Discussion

Spheno-orbital meningiomas (SOM) account for only 4%-9% of all intracranial meningiomas [16]. Due to the penetrative capabilities of SOMs, they can invade local structures, such as the orbit or cavernous sinus. Since these types of tumors can typically infiltrate such vital structures, surgical management can be increasingly difficult. When SOM was first described in the literature, many papers that were published demonstrated discouraging results, a high rate of recurrence led to the recommendation of a conservative approach to treatment [17].

Improvements in the management of brain tumors have led to decreased morbidity and increased survival rates, with the goal being to resect as much tumor as possible without causing any additional harm. However, even with the many great advances in surgical technique and imaging, studies show that the number one reason for brain tumor recurrence is known residual tumor following initial resection leading to increased morbidity and mortality in these patients [18]. Furthermore, even for brain tumors that were considered acceptable for 100% resection preoperatively, there still remains fragments of tumor leftover in patients postoperatively in over 75% of cases. These studies show the importance of having newer surgical methods to more accurately help in total resection of a tumor.

The Raman spectrum of any molecule can be determined by measuring the Raman shifts interacting with the molecule’s chemical bonds and can be used for chemical identification. Chemical differences between tumor and normal tissue create distinguishably different Raman spectra and thus allow for the identification of tissue boundaries [19]. Applications of stimulated Raman histology in brain tissue continue to provide promising results by being able to distinguish normal brain from tumor-cell infiltrated brain with high sensitivity and specificity [20]. Furthermore, studies have shown a significant reduction in the time to completion of pathologic preparation when using stimulated Raman histology versus standard techniques [11].

Three studies published within the last 15 years examined the diagnostic accuracy of stimulated Raman histology in differentiating meningioma from normal tissue; the sensitivity and specificity determined in these studies were 0.98 and 1.00, respectively, with high diagnostic accuracy [12-14]. Koljenovic et al. studied meningioma and normal dura using stimulated Raman histology and noted that large differences exist between the Raman spectra of meningioma and dura due to the high lipid content in the tumor and the high collagen content of the dura. The Raman spectra discriminant analysis yielded an accuracy of 100% in 20 patients, further showing that stimulated Raman histology is a promising intraoperative neurosurgical tool in differentiating meningioma from normal tissue [13]. In this case, stimulated Raman histology allowed us to confirm tumor margins and identify the surrounding normal tissue with a high level of accuracy. When achieving negative margins are needed during oncologic resection, stimulated Raman histology has the advantage of providing fast and reliable feedback to help guide the surgeon while additional pathological data is gathered and processed.

## Conclusions

Our case demonstrates the utility of stimulated Raman histology as an effective tool for differentiating meningioma from normal tissue. Its use as a technique to identify tissue margins can help ensure complete resection of the tumor while preventing iatrogenic injury to adjacent normal structures, thus improving overall accuracy in brain tumor surgeries while circumventing the need for tissue preparation with standard pathology techniques.
